# Detecting Visual Field Worsening From Optic Nerve Head and Macular Optical Coherence Tomography Thickness Measurements

**DOI:** 10.1167/tvst.13.8.12

**Published:** 2024-08-08

**Authors:** Alex T. Pham, Annabelle A. Pan, Chris Bradley, Kaihua Hou, Patrick Herbert, Chris Johnson, Michael Wall, Jithin Yohannan

**Affiliations:** 1 Wilmer Eye Institute, Johns Hopkins University School of Medicine, Baltimore, MD, USA; 2 Malone Center for Engineering in Healthcare, Johns Hopkins University, Baltimore, MD, USA; 3 University of Iowa, Iowa City, IA, USA

**Keywords:** visual field, optical coherence tomography, glaucoma, macula

## Abstract

**Purpose:**

Compare the use of optic disc and macular optical coherence tomography measurements to predict glaucomatous visual field (VF) worsening

**Methods:**

Machine learning and statistical models were trained on 924 eyes (924 patients) with circumpapillary retinal nerve fiber layer (cp-RNFL) or ganglion cell inner plexiform layer (GC-IPL) thickness measurements. The probability of 24-2 VF worsening was predicted using both trend-based and event-based progression definitions of VF worsening. Additionally, the cp-RNFL and GC-IPL predictions were combined to produce a combined prediction. A held-out test set of 617 eyes was used to calculate the area under the curve (AUC) to compare cp-RNFL, GC-IPL, and combined predictions.

**Results:**

The AUCs for cp-RNFL, GC-IPL, and combined predictions with the statistical and machine learning models were 0.72, 0.69, 0.73, and 0.78, 0.75, 0.81, respectively, when using trend-based analysis as ground truth. The differences in performance between the cp-RNFL, GC-IPL, and combined predictions were not statistically significant. AUCs were highest in glaucoma suspects using cp-RNFL predictions and highest in moderate/advanced glaucoma using GC-IPL predictions. The AUCs for the statistical and machine learning models were 0.63, 0.68, 0.69, and 0.72, 0.69, 0.73, respectively, when using event-based analysis. AUCs decreased with increasing disease severity for all predictions.

**Conclusions:**

cp-RNFL and GC-IPL similarly predicted VF worsening overall, but cp-RNFL performed best in early glaucoma stages and GC-IPL in later stages. Combining both did not enhance detection significantly.

**Translational Relevance:**

cp-RNFL best predicted trend-based 24-2 VF progression in early-stage disease, while GC-IPL best predicted progression in late-stage disease. Combining both features led to minimal improvement in predicting progression.

## Introduction

Optical coherence tomography (OCT) plays a major role in managing glaucoma. It often involves imaging the optic nerve head and measuring the retinal nerve fiber layer thickness in the circumpapillary region (cp-RNFL). Thinning of the cp-RNFL can indicate axonal loss owing to pressure-related injury of retinal ganglion cells (GCs) within the optic nerve. Because structural glaucomatous changes are frequently observed before functional glaucomatous changes,^1^ OCT imaging can help with the early detection of disease progression so that therapeutic interventions can be delivered promptly before the onset of vision loss detectable with visual field (VF) testing. However, once cp-RNFL thinning has progressed past the measurement floor, no further structural changes can be observed with OCT imaging.^2^^–^^4^ Hence, the usefulness of optic nerve head OCT imaging in identifying disease progression is limited for eyes with later stages of glaucoma owing to this floor effect.

More recent applications of OCT involve imaging the macula to measure the GC inner plexiform layer (IPL) thickness, because this region has the highest density of retinal GCs^5^^,^^6^ and is known to be affected in early glaucoma.^7^^–^^13^ Prior studies have demonstrated that GC-IPL analysis is at least as accurate as cp-RNFL analysis in predicting VF progression in the early stages of glaucoma.^14^^,^^15^ Moreover, GC-IPL analysis may be more suitable for detecting progression in advanced disease because the floor effect is observed later than in cp-RNFL analysis.^16^^–^^18^

Most studies comparing the role of cp-RNFL and GC-IPL in assessing glaucoma progression have focused on simple statistical approaches such as linear regression.^19^^–^^27^ Exploring a machine learning approach to predict VF worsening based on structural parameters may be beneficial, because machine learning classifiers have shown similar or improved diagnostic accuracy for detecting glaucoma compared to with traditional statistical models.^28^^,^^29^ Machine learning models can also combine features from different sources more complexly than simple statistical models. Therefore, they may provide insight into the clinical usefulness of using cp-RNFL and GC-IPL thickness measurements to detect progression. This factor is especially important because combining cp-RNFL and GC-IPL analyses is considered by some to be the best practice in OCT-based clinical glaucoma assessment.^30^^,^^31^

Our study aimed to compare the diagnostic accuracy of machine learning models using cp-RNFL alone, GC-IPL alone, and cp-RNFL and GC-IPL together to predict VF progression. We also aimed to determine how diagnostic accuracy changes across a broad range of disease severity, because prior work has been limited to only moderate and advanced glaucoma.^32^ Last, we aimed to compare the performance of machine learning models with traditional statistical approaches, such as logistic regression, for modeling VF worsening.

## Methods

The Johns Hopkins University Institutional Review Board approved this study to evaluate retrospective data with a waiver of consent. The study was completed following the tenets of the Declaration of Helsinki and was compliant with the Health Insurance Portability and Accountability Act.

### Data Curation

Eligible patients considered for our study were adults with a glaucoma or glaucoma-related diagnosis seen at the Wilmer Eye Institute Glaucoma Center of Excellence between September 2012 and December 2023. Our dataset consisted of eyes with at least five VF tests, five optic nerve head OCT scans, and five macular OCT scans. Both optic nerve head and macular OCT scans had to be paired uniquely within 1 year of a VF test. If both eyes from the same patient satisfied the inclusion criteria, a single eye was selected randomly for analysis.

### OCT Imaging

All OCT scans were acquired using a CIRRUS HD-OCT (Zeiss, Dublin, CA) instrument. Only reliable OCT scans were included for analysis. Reliable optic nerve head OCT scans were defined as having a signal strength of greater than 6 and average, superior quadrant, and inferior quadrant RNFL thickness measurements between 33 and 150 µm.^33^ Reliable macular OCT scans were defined as having a signal strength of greater than 6 and average GC-IPL thickness measurements between 26 and 100 µm.

The optic nerve head and macular features measurements were based on a 6 mm × 6 mm data cube captured by the Optic Disc/Macular Cube 200 × 200 scan. The optic nerve head and macular OCT scan measurement floor thresholds were defined as three standard deviations below the minimum GC-IPL and the average RNFL thickness values observed in eyes with advanced glaucoma.^34^ Similarly, ceiling thresholds were defined as thickness values three standard deviations above the average GC-IPL and average RNFL thickness observed in healthy eyes.^34^ OCT scans with thickness measurements below the floor or above the ceiling were excluded because they are likely due to imaging artifacts or segmentation errors.

### VF Testing

All VF tests were obtained using a Humphrey Visual Field Analyzer II or III instrument with the SITA Standard, Fast, or Faster testing algorithm and the 24-2 test pattern. Only reliable VF tests were included for analysis. Reliable VF tests were defined as having false-positive rates of less than 15% for all stages of the disease, false-negative rates of less than 25% for suspect or mild glaucoma, and false-negative rates of less than 50% for advanced glaucoma.^35^

VF testing was also used to determine baseline disease stage and progression status. A baseline mean deviation (MD) value greater than −6 dB was defined as suspect or mild glaucoma. Glaucoma suspects were differentiated from mild glaucoma based on suspects having glaucoma hemifield test results “within normal limits.” Meanwhile, a baseline MD worse than −6 dB was defined as moderate or advanced glaucoma.

Both trend-based analysis and event-based analysis of VF worsening were explored.^36^ Trend-based analysis was identified by a statistically significant (α = 0.05) MD rate of change coupled with an MD slope worse than −0.50 dB/year.^37^^–^^40^ The MD slope was calculated using ordinary least squares regression. Event-based analysis was determined using an algorithm similar to, but not identical to, guided progression analysis (GPA) because GPA is a proprietary algorithm, and we did not have access to the database needed to reproduce GPA results. The algorithm we used and its rationale are described in detail by Hou et al.^40^ Briefly, an eye was considered progressing if total deviation values at three or more test points at any location on the 24-2 test pattern were significantly worse (α = 0.05) on three or more postbaseline VFs compared with two baseline VFs (the first two VFs measured for that eye). For simplicity, we will use the term GPA-like to refer to this algorithm.

### Machine Learning Approach to Predicting Glaucoma Progression

We created a training and testing set using a 60%:40% split on the dataset described above. Multiple machine learning algorithms were explored, including random forest classifier, support vector classifier, *K*-nearest neighbors classifier, and naïve Bayes classifier. During the training phase, hyperparameters for each model were optimized using a grid search method with 5-fold cross-validation. The machine learning algorithm with the highest area under the curve (AUC) was considered the best-performing algorithm. Trend-based and event-based evaluations were conducted independently so that different best-performing algorithms could be used for each analysis.

Input features considered for the model included demographic features (age, gender), mean intraocular pressure throughout follow-up, and OCT-derived measurements, such as baseline thickness and rates of change for cp-RNFL features (average RNFL, four quadrant RNFLs, 12 clock-hour RNFLs, and the six sectoral RNFL zones described by Garway-Heath et al.^41^), baseline measurements and rates of change for nerve head features (cup-to-disc ratio, vertical cup-to-disc ratio, disc area, and cup volume), and baseline thickness and rates of change for GC-IPL features (average GC-IPL, minimum GC-IPL, total superior or inferior GC-IPL, and six regional GC-IPLs). We trained a cp-RNFL model using only optic nerve measurements from the training set and a GC-IPL model using the macular measurements from the training set. We used subset feature selection to retain only relevant features to decrease model complexity and model overfitting and improve accuracy.^42^^,^^43^ Specifically, we used greedy forward selection, such as in Nouri-Mahdavi et al.,^32^ which involves starting with an empty set of features and iteratively adding each feature one at a time so that features that improve the AUC are kept, and features that do not improve the AUC are discarded.

### Statistical Approach to Predicting Glaucoma Progression

The statistical model we explored was a multivariate logistic regression. The dependent variable was VF progression status, and the independent variables were derived by performing greedy forward selection as described for the machine learning model. Thus, the final features selected for the statistical model could differ from those used in the machine learning model.

### Analysis of Best-performing Machine Learning and Statistical Model

The primary outcome of this investigation is the AUC of the best-performing machine learning and the statistical model when evaluated using the testing set. For both approaches, cp-RNFL probability predictions were generated using only the optic nerve head data derived from the testing set. GC-IPL probability predictions were generated using only macular data from the testing set. Hence, each cp-RNFL and GC-IPL model from the machine learning and statistical approaches were evaluated with the same eyes. We were also interested in determining whether combining cp-RNFL and GC-IPL information would improve the ability to predict VF worsening. The combined predictions were calculated using the disjunction rule in probability theory:

$$\begin{array}{ccc} & & \;p\left(\mathrm{OC}T_{1}\;\cup \;\mathrm{OC}T_{2}\right)\\ & & \;=p\left(\mathrm{OC}T_{1}\right)+p\left(\mathrm{OC}T_{2}\right)-p\left(\mathrm{OC}T_{1}\;\cap \;\mathrm{OC}T_{2}\right), \end{array}$$

where *p*(OCT_1_), *p*(OCT_2_), *p*(OCT_1_ ∩ OCT_2_), and *p*(OCT_1_ ∪ OCT_2_) represent the probability of VF worsening based on cp-RNFL only, GC-IPL only, cp-RNFL and GC-IPL, and cp-RNFL or GC-IPL, respectively. The probability estimates of the cp-RNFL, GC-IPL, and combined predictions were used to calculate the AUCs, which were compared using DeLong's test.^44^ As a part of our secondary analysis, we stratified the combined testing set by disease severity to see how the AUCs of the cp-RNFL predictions, GC-IPL predictions, and combined predictions change with stages of disease. We also compared AUCs between the machine learning and statistical models within each stratum of disease severity using DeLong's test.^44^ All analyses were completed using R.^45^

### Sensitivity Analysis

We conducted a sensitivity analysis that involved including the fellow eyes from the same patients if the fellow eye met the inclusion criteria. The same patient could not be present in both the training and test set. Afterward, we recalculated the AUCs for the cp-RNFL, GC-IPL, and combined predictions for both the statistical and machine learning models. For the statistical model, we also explored logistic mixed effects modeling with a random effect term to account for both eyes originating from the same patient. Similar to the description above, statistical comparisons between AUCs were performed using DeLong's test.^44^

## Results

Table shows the demographic, VF, and OCT characteristics of patients included in this study. A total of 1541 eyes were included in the study. The median follow-up period for included eyes was 4.74 years, ranging from 3 to 11 years. The training and testing set consisted of 924 and 617 eyes, respectively. The age, gender, race, glaucoma severity, baseline VF measurements, and baseline OCT measurements were similar between the training and testing sets. Eyes included in our study were predominantly from female and Caucasian patients. The majority of eyes had suspect or mild glaucoma. Only a small percentage (approximately 4%) of eyes were identified as worsening using MD slope and GPA-like definitions of progression.

**Table.** Summary of the Demographic, VF, and OCT Characteristics for Training and Test Sets

	Training Set (*n* = 924)	Test Set (*n* = 617)
Mean age ± SD, years	64 ± 14	64 ± 14
Gender		
Male	381 (43)	238 (39)
Female	543 (57)	379 (61)
Race		
White	599 (64)	402 (65)
Black	234 (27)	153 (25)
Asian	47 (5.4)	27 (4.4)
Other	33 (2.9)	27 (4.4)
Unknown	11 (1.2)	8 (1.3)
Glaucoma Severity		
Suspect	364 (39)	243 (39)
Mild	419 (45)	280 (45)
Moderate	103 (11)	69 (11)
Advanced	38 (4.1)	25 (4.1)
VF Characteristics		
Mean MD ± SD, dB	–2.93 ± 4.02	–3.01 ± 4.23
Mean PSD ± SD, dB	3.30 ± 2.85	3.41 ± 2.99
OCT Characteristics		
Mean RNFL ± SD, µm	81.20 ± 13.79	81.10 ± 13.14
Mean CDR ± SD	0.58 ± 0.19	0.58 ± 0.20
Mean GC-IPL ± SD, µm	70.54 ± 11.48	70.53 ± 11.59
No. MD slope progressors	38 (4.1)	25 (4.1)
No. GPA-like progressors	44 (4.8)	32 (5.2)

CDR, cup-to-disc-ratio; PSD, pattern standard deviation.

Values are mean ± standard deviation or number (%).

**Figure 1.**
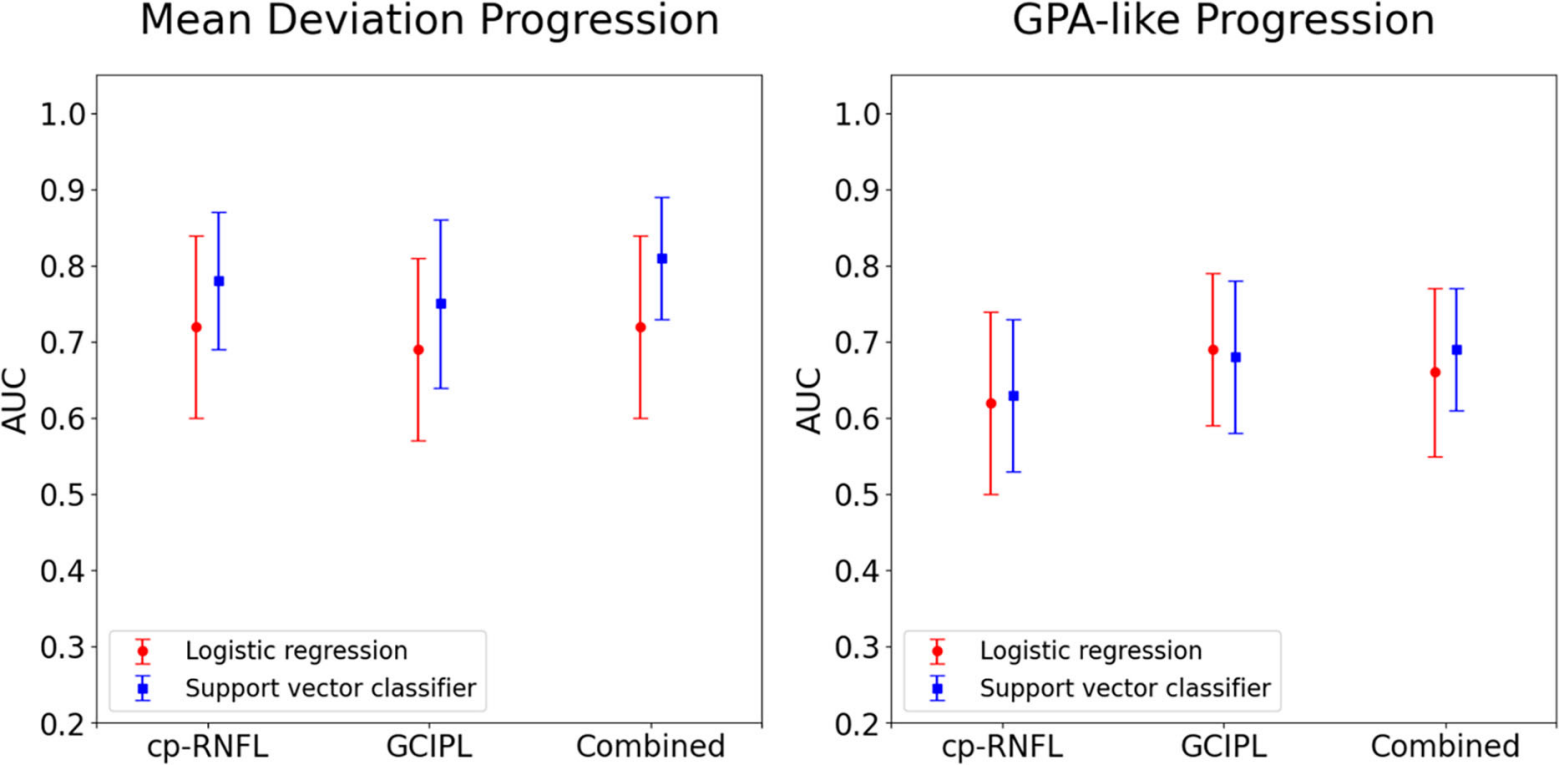
Overall performance (defined by the AUC and the 95% CI) for best-performing statistical and best-performing machine learning models when using cp-RNFL, GC-IPL, or both (combined) to predict VF progression. True labels for VF progression were defined by trend-based (MD slope shown on the left) and event-based (GPA shown on the right) methods.

Support vector classifiers were the best-performing machine learning models for trend- and event-based analysis. Figure 1 shows the AUCs of the statistical and machine learning models for detecting glaucoma progression with ground truths defined by MD slope or GPA-like.

For MD slope progression, the logistic regression AUCs were 0.72 (95% confidence interval [CI], 0.60–0.84), 0.69 (95% CI, 0.56–0.81), and 0.73 (95% CI, 0.61–0.85) for the cp-RNFL, GC-IPL, and combined predictions, respectively. The support vector classifier AUCs were 0.78 (95% CI, 0.69–0.86), 0.75 (95% CI, 0.64–0.86), and 0.81 (95% CI, 0.73–0.89) for the cp-RNFL, GC-IPL, and combined predictions, respectively. The support vector classifier had similar AUCs for cp-RNFL (*P* = 0.48), GC-IPL (*P* = 0.45), and the combined predictions (*P* = 0.24) when compared with the logistic regression. Although combining cp-RNFL and GC-IPL predictions resulted in a slightly higher AUC than either alone, these differences were not statistically significant for the logistic regression (combined vs. cp-RNFL, *P* = 0.85; combined vs. GC-IPL, *P* = 0.27) and support vector classifier (combined vs. cp-RNFL, *P* = 0.21; combined vs. GC-IPL, *P* = 0.13).

For GPA-like slope progression, the logistic regression AUCs were 0.62 (95% CI, 0.50–0.75), 0.69 (95% CI, 0.59–0.79), and 0.66 (95% CI, 0.55–0.76) for the cp-RNFL, GC-IPL, and combined predictions, respectively. The support vector classifier AUCs were 0.63 (95% CI, 0.53–0.73), 0.68 (95% CI, 0.58–0.78 and 0.69 (95% CI, 0.61–0.77), respectively. Compared with the logistic regression, the support vector classifier had similar AUCs for cp-RNFL (*P* = 0.97), GC-IPL predictions (*P* = 0.90), and the combined predictions (*P* = 0.65). For both models, AUCs of cp-RNFL predictions were similar to GC-IPL predictions. Combining the cp-RNFL and GC-IPL predictions did not result in a higher AUC than cp-RNFL and GC-IPL alone.

**Figure 2.**
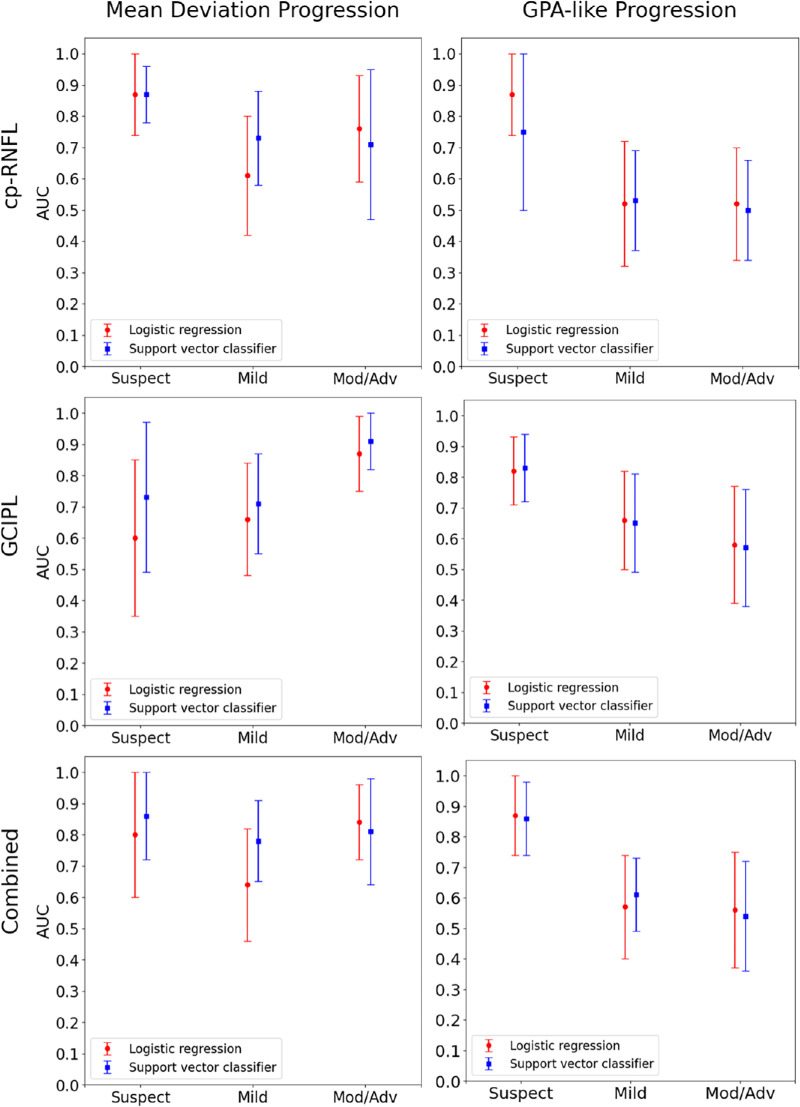
Performance (defined by the AUC and the 95% CI) for the best-performing statistical and machine learning models, stratified by disease severity, when using cp-RNFL, GC-IPL, or both (combined) to predict VF progression. True labels for visual progression were defined by trend-based (MD slope shown on the left) and event-based (GPA shown on the right) methods.

Figure 2 shows the performance of the logistic regression and support vector classifiers stratified by disease severity. For MD slope progression, cp-RNFL predictions from the support vector classifier and logistic regression resulted in the highest AUCs for eyes with suspect glaucoma, and the AUCs decreased with increasing disease severity. In contrast, GC-IPL predictions from the support vector classifier yielded the highest AUCs for eyes with moderate or advanced glaucoma and the lowest AUCs for suspect or mild disease. However, these trends across disease severity were not statistically significant. The AUCs for the statistical and machine learning models were statistically similar regardless of disease stage and the respective prediction types.

For GPA-like progression, Figure 2 demonstrates that cp-RNFL, GC-IPL, and the combined predictions resulted in the highest AUCs in eyes with suspect glaucoma. The AUCs generally decreased with increasing disease severity. For the logistic regression, the AUCs were significantly different between suspect and moderate or advanced disease for cp-RNFL (*P* < 0.001), GC-IPL (*P* = 0.04), and the combined predictions (*P* = 0.01). These differences between suspect and moderate or advanced disease were also significant for the support vector classifier when using GC-IPL and the combined predictions, but not when using cp-RNFL predictions. Like the MD progressors, the AUCs between the logistic regression and support vector classifiers were statistically similar across each disease stage and respective prediction type.

**Figure 3.**
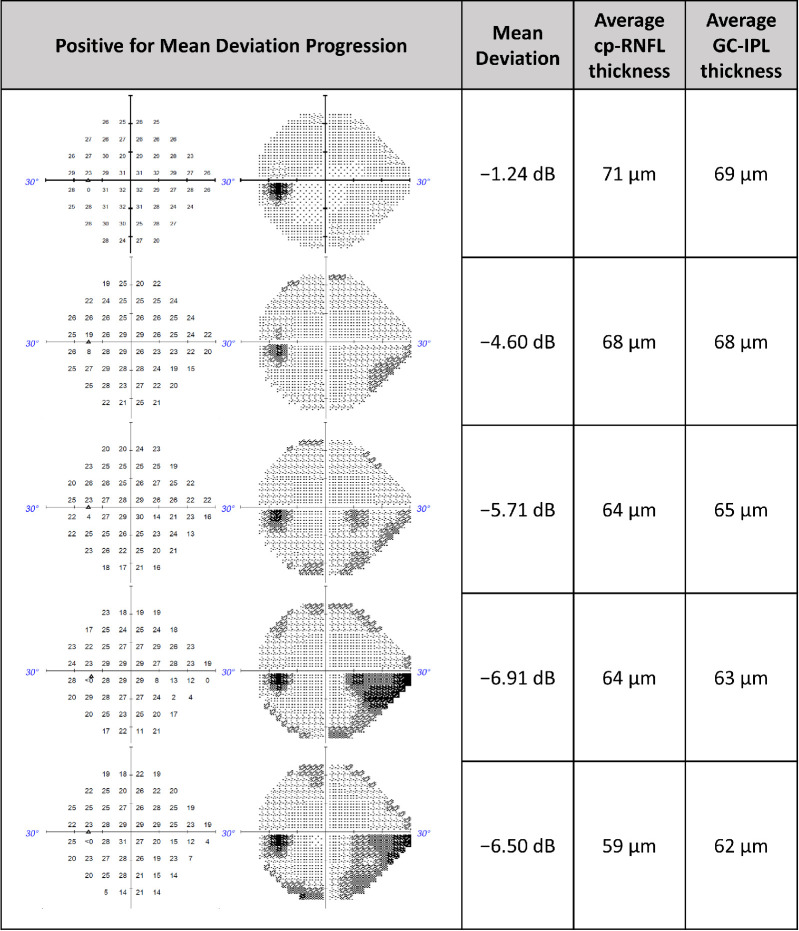
Representative clinical case in which machine learning and statistical models predicted MD progression when using cp-RNFL thickness, GC-IPL thickness, and both (combined).

**Figure 4.**
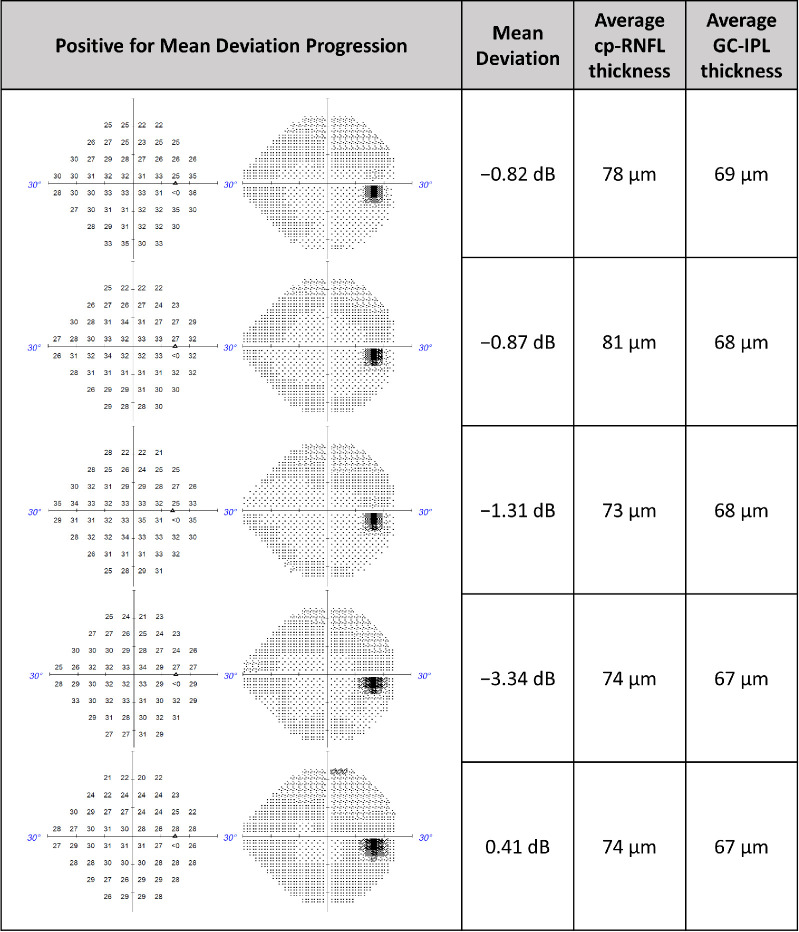
Representative clinical case in which machine learning and statistical models predicted no MD progression when using cp-RNFL thickness, GC-IPL thickness, and both (combined).

Figure 3 illustrates a series of five VF tests in an eye classified as a progressor by the cp-RNFL, GC-IPL, and combined predictions while using MD slope as the ground truth. The corresponding MD and the paired cp-RNFL and GC-IPL measurements are also shown. Figure 3 demonstrates that, as the VF worsens, corresponding structural thinning can be detected in the cp-RNFL and GC-IPL thickness measurements. Meanwhile, Figure 4 illustrates a series of five VF tests in an eye classified as not progressing by the cp-RNFL, GC-IPL, and combined predictions while using MD slope as the ground truth. Similarly, the corresponding MD, cp-RNFL, and GC-IPL measurements demonstrate concordance between the functional and structural parameters.

**Figure 5.**
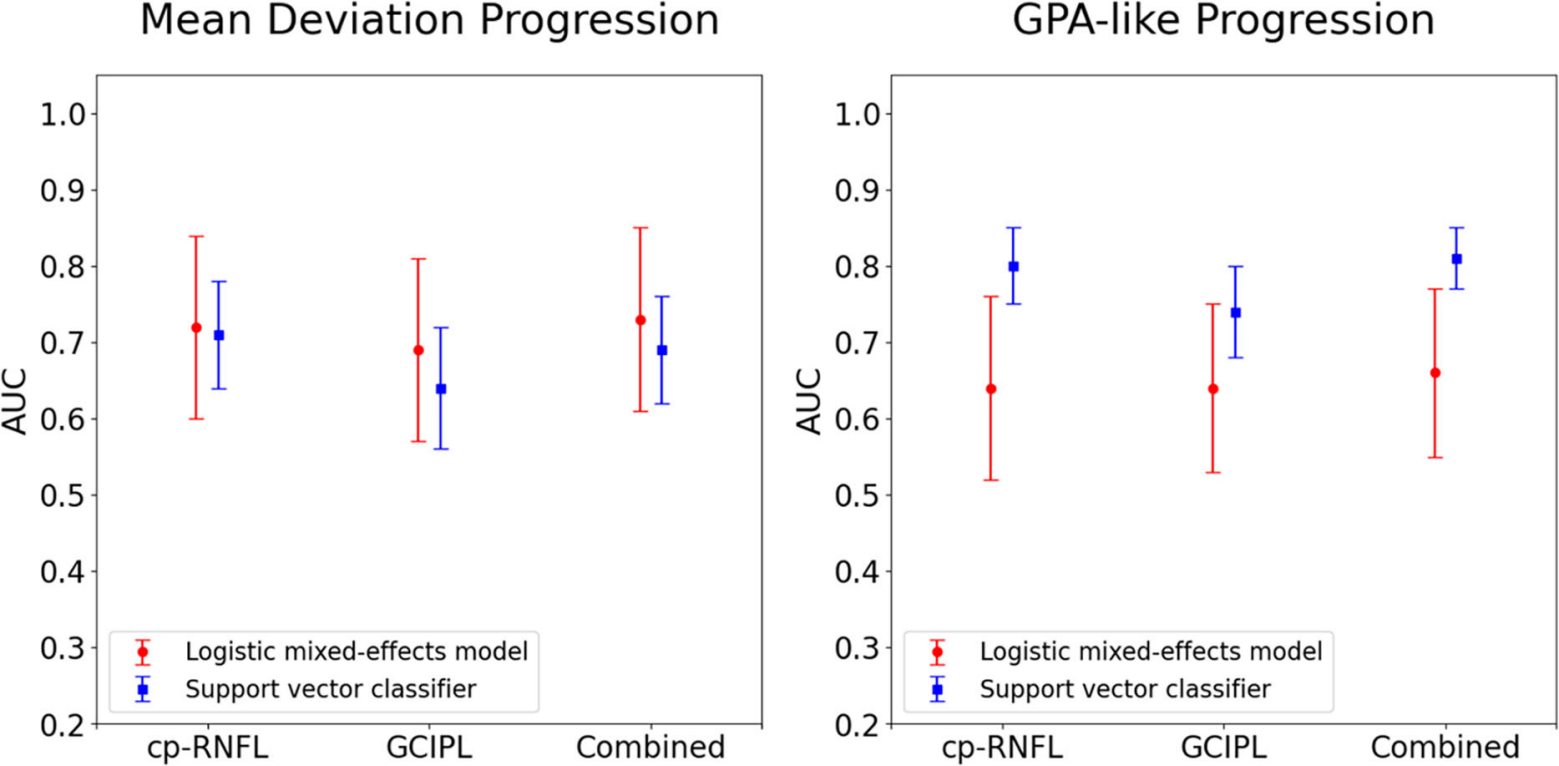
Sensitivity analysis for overall performance (defined by the AUC and the 95% CI) for best-performing statistical and best-performing machine learning models when using cp-RNFL, GC-IPL, or both (combined) to predict VF progression. True labels for VF progression were defined by trend-based (MD slope shown on the left) and event-based (GPA shown on the right) methods.

Our sensitivity analysis involved including fellow eyes, if eligible, from patients, and the results are shown in Figure 5. The training and testing set consisted of 1387 and 966 eyes, respectively. The best-performing statistical model was the mixed effects model. Figure 5 presents trends similar to our original analysis, as shown in Figure 1. The mixed effects model and support vector classifier performed similarly for cp-RNFL, GC-IPL, and the combined predictions when using MD slope for progression. However, the support vector classifier performed better than the mixed effects model for cp-RNFL (*P* = 0.02), GC-IPL (*P* = 0.15), and the combined predictions (*P* = 0.01) when using GPA-like definitions of progression.

## Discussion

This study assessed the ability of statistical and machine learning models to predict VF worsening using cp-RNFL and GC-IPL thickness measurements. We found that using cp-RNFL and GC-IPL resulted in a statistically similar performance when predicting trend-based and event-based 24-2 VF progression among all eyes overall. Combining cp-RNFL predictions with GC-IPL predictions led to a minimal improvement in predictive performance, and differences were not statistically significant. When stratifying by disease severity, we found that cp-RNFL predictions performed best in suspect glaucoma, while GC-IPL predictions performed best in moderate or advanced disease for trend-based VF progression. For event-based VF progression, predictive performance decreased with increasing disease severity for all conditions.

When looking at overall performance, we found that cp-RNFL analysis had similar AUCs to GC-IPL analysis for predicting 24-2 VF worsening for both trend-based and event-based progression. This pattern was still present after our sensitivity analysis, including eligible fellow eyes per patient. In previous studies comparing rates of cp-RNFL thinning with GC-IPL, Leung et al.^25^ found the rate of global cp-RNFL thinning to be faster than the rate of global GC-IPL thinning (−1.53 vs. −0.81 µm/year) in glaucomatous eyes. Hammel et al.^22^ also found that the rate of global cp-RNFL thinning after normalizing for the dynamic range was significantly faster than the rate of global GC-IPL thinning (−1.7% vs −1.3% per year; *P* < 0.001) in glaucomatous eyes. When cp-RNFL and GC-IPL thinning rates are used to predict VF progression, our findings are consistent with those of prior studies. Hou et al. found that although glaucomatous eyes with cp-RNFL thinning had a slightly higher hazard ratio than GC-IPL thinning (3.66 vs. 3.48), both were similarly predictive when used as indicators of VF progression defined by the Early Manifest Glaucoma Trial criteria.^19^^,^^46^ When using trend-based analysis, Lee et al. found that global cp-RNFL and GC-IPL thinning rates had statistically similar AUCs for predicting glaucoma progression (0.68 vs. 0.79; *P* = 0.19).^21^

Compared with trend-based progression, we found that cp-RNFL predictions, GC-IPL, and the combined predictions had lower performance in event-based progression. A possible reason for this discrepancy in performance between trend-based and event-based progression is the inability to detect spatial relationships when using global rates of cp-RNFL or GC-IPL thinning. We did include regional and sectoral cp-RNFL or GC-IPL thinning rates, but these thinning rates may not have enough resolution to detect small changes in individual VF test points.

Stratifying the analysis by disease severity, we found that cp-RNFL analysis had the best predictive performance for trend-based VF progression in suspect glaucoma and the worst in moderate/advanced glaucoma. We could not find a statistically significant difference between the stages of disease severity, but this may be due to the limited sample size of progressors in moderate/advanced. Nonetheless, such a trend is expected owing to the floor effect observed with OCT imaging.^3^^,^^4^^,^^47^ As disease severity increases, the cp-RNFL thins to a point where no further structural changes can be detected. Thus, predicting VF worsening using cp-RNFL in eyes with late-stage disease is difficult.

Conversely, we found that GC-IPL analysis had the best predictive performance for trend-based VF progression in moderate or advanced glaucoma and worse performance in suspect or mild glaucoma. Similar to the cp-RNFL analysis, we did not find a statistically significant difference between the stages of disease severity, perhaps owing to the limited sample size. Our findings align with previous studies that have suggested that GC-IPL analysis is more beneficial than cp-RNFL analysis in the later stages of glaucoma.^21^^,^^22^^,^^32^^,^^48^ Similar to our study, Nouri-Mahdavi et al.^32^ found that a machine learning model using GC-IPL features had a substantially higher AUC than when using cp-RNFL features (0.80 [95% CI, 0.69–0.91] vs. 0.61 [95% CI, 0.46–0.76]) in eyes with moderate to advanced glaucoma. This difference in performance between GC-IPL and cp-RNFL analysis in late-stage glaucoma is likely because GC-IPL has a lower floor than cp-RNFL.^16^^–^^18^

When combining cp-RNFL predictions with GC-IPL predictions, we observed similar or slightly improved (although not statistically significant) performance in predicting 24-2 VF worsening compared with either alone. Nouri-Mahdavi et al.^32^ also found that using both cp-RNFL and GC-IPL features did not result in a substantially higher AUCs than using either cp-RNFL or GC-IPL features alone in eyes with moderate to advanced glaucoma. In contrast, Wu et al.^49^ found that integrating both cp-RNFL and GC-IPL parameters into a single layer (capturing the parapapillary region and macular region in a single wide-field OCT scan) was able to detect more eyes with disease progression at an earlier time compared with cp-RNFL or GC-IPL alone for trend-based analysis. It is important to note that, although the combined analysis in their study revealed that a larger proportion of eyes that had VF worsening (they used Early Manifest Glaucoma Trial criteria to define worsening^46^), the hazard ratio of combined thinning was not higher than cp-RNFL thinning after adjusting for covariates such as age and baseline cp-RNFL/GC-IPL thickness (4.6 [95% CI, 1.5–14.0] vs. 4.9 [95% CI, 1.42–16.9]). Another possible reason for the discrepancy between our findings and theirs is the differences between study populations. Because the majority of eyes included in our study are suspect or mild glaucoma, the benefit of macular OCT in late-stage disease for detecting VF progression may not be reflected in the overall AUC. Figure 2 demonstrates that the performance of the combined predictions in moderate or advanced glaucoma is higher than that of the cp-RNFL predictions, although we did not find statistical significance.

Generally, we found that our best-performing machine learning model performed similarly to the statistical model at predicting VF progression using structural parameters. Our finding is consistent with Nouri-Mahdavi et al.,^32^ who reported similar AUCs between an elastic net regression model and a naïve Bayes classifier. This result suggests that it may be more practical to use statistical models over machine learning models to predict progression because they offer similar performance while requiring fewer computational resources. However, our sensitivity analysis (with a larger dataset including fellow eyes) suggests that machine learning models could have improved performance compared with traditional statistical models when using a more robust dataset and when event-based analysis is used for progression. In a prior study, we investigated using a more sophisticated machine learning approach, specifically a gated transformer network, for predicting 24-2 VF progression using a larger dataset of 4211 eyes with longitudinal optic nerve head OCT scans.^40^ We found that the deep learning model significantly outperformed other statistical models, such as mixed effects models, using the same optic nerve head OCT inputs. We did not investigate a deep learning model in this study owing to the smaller dataset of eyes with macular imaging. Thus, further work is needed to examine whether applying a similar approach to macular OCT inputs would produce similar results once macular OCT imaging becomes more prevalent, and there are a more significant number of patients with longitudinal macular OCT imaging.

Our study has several strengths and limitations. Unfortunately, because macular OCT imaging was adopted in our clinic later than optic nerve head OCT imaging, there are fewer eyes with longitudinal macular imaging. To keep the number of eyes similar in both training sets, we did not use the entirety of our longitudinal optic nerve head OCT dataset, leaving the possibility that our machine learning model's performance could be better, especially because we did find improved performance with deep learning models over traditional statistical models using similar OCT inputs in our prior study using cp-RNFL data only.^40^ Our study also includes a broad range of disease severities, representative of real clinical circumstances. However, the number of progressing eyes in the training data was modest, and the majority of progressing eyes were suspect or mild glaucoma. Rather than creating separate cp-RNFL and GC-IPL models and combining their predictions, another approach that may produce different findings is building a single model taking both cp-RNFL and GC-IPL as inputs. Our study included a small percentage of SITA Faster VF tests in both the training and test sets, which may affect our trend- and event-based progression analysis. However, most eyes included in this study were suspect and mild glaucoma. VF measurements obtained with SITA Faster have been shown to be similar to SITA Standard, at least in the early stages of glaucoma.^50^^,^^51^ For VF progression, our analysis involved global metrics for trend-based progression and GPA for event-based progression. However, more recent methods exist to define progression, such as permutation of pointwise linear regression, which we did not use in this study.^52^ We also did not examine the location of the VF defects in our sample or include 10-2 VF tests, which may affect our findings involving macular data. Owing to their traditional use in the literature, we investigated logistic regression and logistic mixed effects modeling. Other, more sophisticated statistical models, such as structural equation models, may be more appropriate. We used greedy forward selection during feature selection owing to its previous use.^32^ Limitations of this approach are that we did not protect against false discovery rate, and greedy forward selection does not reveal the optimal set of features because only one feature is added at a time. Last, we did not control for coexisting eye diseases, such as cystic macular edema, which can affect the macular OCT thickness measurements.

In summary, we demonstrate that 24-2 VF worsening defined by trend-based analysis can be predicted with modest accuracy using longitudinal cp-RNFL and GC-IPL thickness measurements. The ability to predict VF worsening in early glaucoma was best detected using cp-RNFL features. In contrast, the ability to predict VF worsening in late-stage glaucoma was best detected using GC-IPL features. Combining cp-RNFL and GC-IPL predictions did not significantly improve the ability to predict VF worsening. Meanwhile, changes in structural features had lower predictive performance at predicting event-based VF change.
